# Mental health service use after the Van Earthquake: evidence from a quasi-experimental difference-in-differences study

**DOI:** 10.1186/s12889-026-27614-8

**Published:** 2026-05-18

**Authors:** Demet Topal Koç, Gabriel Picone, Bradley Kamp

**Affiliations:** Department of Health Management, Kırklareli University, Kırklareli, Türkiye

**Keywords:** Earthquake exposure, Mental health service use, Difference-in-differences, Natural disasters, I12, I18, Q54, C21

## Abstract

This study examines the long-run effects of disaster exposure on mental health service utilization using quasi-experimental difference-in-differences and event-study design. Using nationally representative Türkiye Health Survey data (2008–2022), we analyze changes in mental health service use following the 2011 Van earthquake. We document a delayed but sustained increase in utilization of approximately 3–4% points, peaking in 2016–2019 and attenuating by 2022. Heterogeneity analyses show larger responses among men and single individuals, while utilization among women remains comparatively lower. Robustness checks, including alternative control groups and wild-cluster bootstrap inference, support the validity of the empirical design. The findings highlight that post-disaster mental health service use may evolve gradually over time, underscoring the importance of long-term recovery planning in middle-income, high-seismic-risk settings.

## Introduction

Natural disasters disrupt not only physical infrastructure but also individual well-being and patterns of health-seeking behavior. Earthquakes, in particular, can generate psychological distress that persists long after the immediate shock, potentially shaping how individuals interact with health systems over time. While a large economic literature has examined the macroeconomic, labor market, and demographic consequences of disasters, far less is known about how disaster exposure translates into the use of formal mental health services, especially beyond the immediate aftermath and outside high-income country contexts.

This study examines how exposure to a major earthquake is associated with changes in mental health service utilization in Türkiye, a country characterized by high seismic risk and an evolving mental health care system. Using seven waves of nationally representative Türkiye Health Survey data spanning 2008–2022, we apply a difference-in-differences and event-study framework to analyze utilization patterns following the 2011 Van earthquake. We document a delayed but sustained increase in mental health service use in the affected region relative to nearby comparison regions, with differences emerging several years after the disaster and attenuating by 2022.

The delayed timing of the observed response suggests that behavioral reactions to large shocks may unfold gradually rather than immediately. Factors such as stigma, delayed recognition of psychological need, and barriers to access may postpone help-seeking even when underlying distress is substantial. From a public health perspective, this dynamic adjustment underscores the importance of long-term recovery planning and sustained mental health system capacity, particularly in settings where access to care is uneven.

This study makes three main contributions to the disaster and mental health literature. First, it provides micro-level evidence on the medium- to long-run association between disaster exposure and formal mental health service utilization in an upper-middle-income country, moving beyond the short-run focus of much existing work. Second, by adopting a dynamic difference-in-differences design, it documents delayed and persistent utilization patterns that would be obscured in static post-disaster comparisons. Third, it situates these patterns within a behavioral framework, emphasizing how social stigma, behavioral inertia, and delayed help-seeking may shape observed service use over time. While our quasi-experimental design strengthens causal interpretation relative to descriptive approaches, we interpret the estimates as changes in observed utilization associated with earthquake exposure rather than definitive causal effects.

Before proceeding, it is important to clarify the study’s scope. This paper does not aim to identify a fully isolated causal effect of the Van earthquake at the individual exposure level. Given data availability, the analysis is intentionally conducted at the NUTS-2 regional level and is designed to document post-disaster regional patterns in observed mental health service utilization. Accordingly, the estimates should be interpreted as associations capturing post-earthquake utilization dynamics, rather than as definitive causal effects net of all concurrent national trends.

Our identification strategy compares trends in mental health service utilization in the earthquake-affected region with those in nearby unaffected regions. A potential limitation of this approach is the presence of regional spillovers, as economic disruption, population movements, and psychological stress may extend beyond the directly affected area. Accordingly, our estimates capture regional differences in observed utilization rather than strictly spillover-free effects. We frame our contribution with this limitation in mind, emphasizing the dynamic evolution of post-disaster mental health service use under realistic regional interdependencies.

## Literature review and theoretical framework

The literature on disasters and mental health can be broadly grouped into three strands. The first documents short-run psychological distress following disasters, typically measured through self-reported symptoms such as anxiety, depression, or post-traumatic stress. The second focuses on vulnerability, inequality, and access, emphasizing how demographic and structural factors shape differential exposure and recovery. A third, more limited strand employs quasi-experimental designs to examine changes in mental health outcomes or service utilization.

Within economics, disasters have been extensively studied for their macroeconomic and regional impacts, including effects on infrastructure, labor markets, supply chains, and migration (Cavallo and Noy [1]; Botzen et al. [2]; Best and Burke [3]; Cavallo et al. [4]; Arto et al. [5]; Carvalho et al. [6]; Higashi [7]; Basile et al. [8]). Economists have also long recognized the importance of mental health for labor productivity, human capital accumulation, and long-term social mobility. Early quasi-experimental evidence suggests that disaster exposure can worsen mental health outcomes, with particularly strong effects among socially vulnerable groups (Zahran et al. [9]), consistent with vulnerability theory [10]. Related work has examined the implications of major earthquakes for mental health policy and institutional responses (Aker [11]; Harada et al. [12]).

Recent clinical studies further document substantial psychological distress following large-scale disasters. Evidence from the 2023 Kahramanmaraş earthquakes indicates elevated anxiety, depression, and trauma-related symptoms across population groups, including women, adolescents, and indirectly exposed individuals [13–15]. While these studies provide important insights into the psychological burden of disasters, they primarily focus on symptoms rather than observed use of formal mental health services.

Complementary evidence from Eastern Europe highlights how disasters may simultaneously increase psychosocial needs and expose institutional gaps in access to care. Studies from Romania and Serbia document heightened fear, anxiety, and reliance on informal support systems in disaster-affected communities, alongside limited preparedness of formal mental health services [16, 17]. Together, this literature underscores the role of structural inequalities, access barriers, and behavioral factors, such as stigma and perceived need, in shaping post-disaster help-seeking behavior.

To interpret these patterns, this study draws on three interrelated conceptual perspectives. Vulnerability theory emphasizes that disasters do not affect populations uniformly, with gender, age, and marital status shaping both exposure and recovery [18]. The concept of state–society synergy highlights how institutional capacity and civic engagement jointly influence post-disaster responses [19, 20], as illustrated by evidence from Türkiye following earlier earthquakes (Jalali [21]). Finally, behavioral economics provides insight into why help-seeking may be delayed, emphasizing behavioral inertia, stigma, and imperfect recognition of psychological need under stress.

While much of the early disaster literature focused on short-run psychological distress, a growing body of economic research has documented that earthquake exposure may generate persistent and multidimensional effects, extending beyond immediate health outcomes to long-term human capital formation, regional development, and institutional capacity.

In particular, quasi-experimental evidence from Japan following the 2011 Great East Japan Earthquake provides some of the most rigorous documentation of persistent mental health impacts at the population level, with several studies showing that psychological distress remains elevated four years after the disaster and beyond [12, 22–24].

Extending beyond contemporaneous mental health outcomes, several studies exploit earthquakes as natural experiments to examine early-life exposure and long-run human capital consequences. Recent evidence from Myanmar exploiting two major earthquakes as natural experiments documents substantial long-run human capital consequences. Early-life exposure, whether in utero or during early childhood, is associated with a higher probability of functional limitations in adulthood, including impairments in vision, hearing, mobility, and cognitive functioning. Affected cohorts also attain lower levels of education, with earthquakes widening pre-existing gender gaps in schooling, suggesting that girls’ human capital accumulation is disproportionately constrained [25]. Comparable quasi-experimental evidence from Nepal (1988 earthquake) and China (Tangshan 1976) shows that individuals exposed in utero are significantly less likely to complete middle and high school, with more pronounced losses among socioeconomically disadvantaged groups and females [26, 27]. Collectively, these findings support a broader mechanism in low-income settings whereby early-life seismic exposure generates persistent reductions in education and health, thereby reinforcing structural inequalities over the long run [25, 27–29].

At a broader structural level, disaster exposure has also been shown to reshape regional development trajectories through capital reallocation and differential recovery dynamics. Evidence from India indicates that natural disasters, including earthquakes, generate persistent spatial reallocation of capital. A nationwide study finds substantial and long-lasting declines in foreign direct investment (FDI) inflows to disaster-affected districts. Importantly, more than two-thirds of the observed investment losses reflect intra-national relocation rather than global withdrawal, with capital shifting toward safer and more developed states. This reallocation pattern contributes to widening regional disparities and long-run spatial divergence in economic activity [30]. Complementary seismic risk assessments for India’s northeast and Himalayan regions suggest that the combination of high social vulnerability and strong seismic exposure implies considerable future economic and human loss potential in the absence of substantial resilience investments. These findings underscore that disaster risk can operate as a structural constraint on regional development trajectories, particularly in socioeconomically vulnerable areas [31, 32].

Using global panel data on physical ground shaking over 1973–2015, Lackner [33] shows persistent long-run output effects, estimating that a typical earthquake is associated with lower GDP per capita even several years after the shock, with losses concentrated in low- and middle-income countries. Complementing this cross-country evidence, subnational work for China finds that both moderate and strong earthquakes reduce prefecture-level GDP per capita in the long run, with effects mediated through household savings, fixed investment, and innovation, and mitigated by local fiscal autonomy and social capital [34].

At the household level, long-term welfare effects are heterogeneous. Evidence from rural Indonesia suggests that repeated large earthquakes initially generate welfare losses but may be followed by higher consumption and improved welfare outcomes six to twelve years later, particularly where reconstruction and external assistance support asset rebuilding and infrastructure recovery [35, 36]. Similarly, in Christchurch, New Zealand, post-earthquake reconstruction was associated with sustained productivity gains, especially within the construction sector, lasting five to eight years after the disaster [37]. However, such recovery-driven gains are typically observed in contexts characterized by strong institutional capacity and coordinated reconstruction efforts. In contrast, poorer or institutionally weaker regions more often experience persistent welfare losses and heightened poverty risks rather than economic convergence [33, 36, 38].

Taken together, this expanding literature documents persistent health, human capital, and regional development consequences of earthquake exposure across diverse institutional settings. However, despite the well-documented psychological and macroeconomic effects, little causal evidence exists on how disaster exposure translates into the medium- and long-run utilization of formal mental health services, particularly in nationally representative middle-income contexts. By focusing on the dynamic evolution of formal mental health utilization rather than self-reported symptoms or aggregate macroeconomic outcomes, this study bridges the gap between clinical distress evidence and broader economic persistence research.

The remainder of the paper is organized as follows. "Data" section describes the data, "Methodology" section outlines the empirical strategy, "Findings and Results" section presents the results, and "Discussion" section concludes.

### Data

This study uses microdata from the Türkiye Health Survey (THS), a nationally representative repeated cross-sectional survey conducted by the Turkish Statistical Institute (TÜİK). We combine seven survey waves collected in 2008, 2010, 2012, 2014, 2016, 2019, and 2022. The first two waves precede the 2011 Van earthquake, while subsequent waves capture post-earthquake periods, with the 2022 wave analyzed separately given the potential confounding effects of the COVID-19 pandemic.

The unit of analysis is the individual respondent. Our outcome of interest is mental health service utilization, measured by a binary indicator capturing whether the respondent reported visiting a psychologist or psychiatrist within the past year. All analyses are conducted at the NUTS-2 regional level, consistent with data availability. The use of NUTS-2 regions reflects a structural data constraint rather than a modeling choice. Due to confidentiality restrictions, the Türkiye Health Survey does not provide province-level identifiers, geocoded locations, or distance-to-epicenter measures. As a result, treatment assignment necessarily aggregates directly affected and indirectly exposed areas within the same NUTS-2 region, which may introduce measurement error and attenuate estimated effects. This limitation motivates a cautious interpretation of the results and reinforces our focus on regional utilization patterns rather than fine-grained causal identification.

**Fig. 1:**
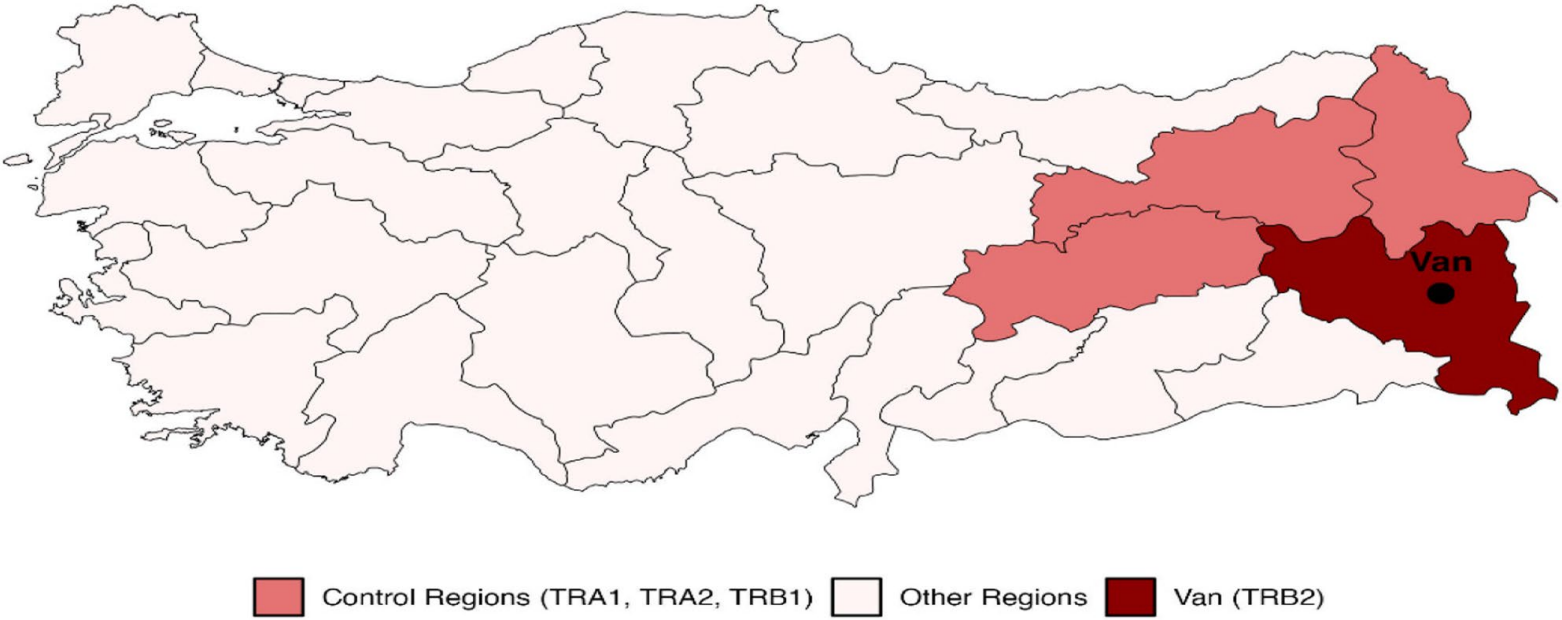
NUTS2 level map of Türkiye. Source: Map generated using shapefiles from TRmaps [39]. Notes: Southern adjacent regions (TRC1–TRC3) are excluded due to large post-2011 refugee inflows that substantially altered local healthcare demand and utilization patterns

Figure 1 presents the NUTS-2 regional map of Türkiye and highlights the treatment and comparison regions. The treatment region is TRB2, which includes the provinces of Van, Muş, Bitlis, and Hakkari. Comparison regions include TRA1 (Erzurum, Erzincan, Bayburt), TRA2 (Ağrı, Kars, Iğdır, Ardahan), and TRB1 (Malatya, Elazığ, Bingöl, Tunceli). These regions were selected based on geographic proximity and similar pre-earthquake socioeconomic characteristics, including income distribution, educational attainment, and age composition.

Regional identifiers in the THS microdata are provided through the official NUTS-2 classification (IBBS2), which takes values from 1 to 26. These identifiers are assigned directly by TÜİK, and no additional regional matching or imputation is performed.

The THS includes detailed demographic and socioeconomic information, allowing us to control for individual characteristics that may influence mental health service use. These covariates include gender, age, income, education, and marital status (see Appendix Table 2 for summary statistics). The sharp increase in income non-response after 2011 occurs simultaneously in both treatment and control regions, suggesting a survey-related measurement change rather than a behavioral response to the earthquake. However, as detailed survey documentation on questionnaire changes is not available to the authors, this interpretation remains tentative. The main analyses are estimated without applying survey sampling weights. While the Türkiye Health Survey follows a complex sampling design, information on primary sampling units (PSUs) is not available in the restricted-use microdata accessed for this study. As a result, it is not possible to explicitly account for PSU-level clustering. Given that treatment is defined at the NUTS-2 regional level, standard errors are clustered at the region level to account for within-region correlation over time. Due to confidentiality restrictions, the Türkiye Health Survey does not provide geocoded household locations or sub-regional identifiers. As a result, it is not possible to construct distance-to-epicenter or intensity-weighted exposure measures, and treatment is necessarily defined at the NUTS-2 regional level.

To assess the sensitivity of the results to specific survey years, we estimate specifications that distinguish the immediate post-earthquake period from later recovery phases and treat the 2022 wave separately. These design choices reflect institutional and contextual factors rather than assumptions about behavioral responses.

## Methodology

Baseline two-period difference-in-differences. We begin with a standard two-period difference-in-differences specification to assess whether there is an immediate change in mental health service utilization following the 2011 Van earthquake. Specifically, we compare outcomes in the treatment and control regions using a narrow time window covering the pre-earthquake period (2010) and the immediate post-earthquake period (2012–2014), excluding later years to avoid contamination from longer-run trends and the COVID-19 pandemic. Estimates from this specification show no statistically significant difference in utilization between the treated and control regions in the immediate post-disaster period, suggesting the absence of a short-run mechanical response. These results are reported in Appendix E (Table (12) and serve as a baseline check motivating the use of a dynamic difference-in-differences framework to examine how treatment effects evolve.

We employ a difference-in-differences (DiD) framework to examine changes in mental health service utilization associated with exposure to the 2011 Van earthquake. Our baseline specification takes the following form:

1 $$\begin{aligned}\:{Y}_{i,r,t}=\alpha\:&+\sum\:_{t\ne\:2008}{\beta\:}_{t}\left({treatment}_{r}\times\:{year}_{t}\right)\\&+{\lambda\:}_{r}+{\delta\:}_{t}+\gamma\:{X}_{i,r,t}+\epsilon_{i,r,t}\end{aligned}$$

where $\:{Y}_{i,r,t}$ is a binary indicator equal to one if individual $\:i\:$ in region $\:r\:$ and survey year $\:t\:$ reports having used mental health services within the past year. $\:Treatmen{t}_{r}$ identifies the earthquake-affected region, and $\:Yea{r}_{t}$ denotes survey-year indicators, with 2008 serving as the reference period. The vector $\:{X}_{i,r,t}$ includes individual-level covariates, gender, marital status, age group, education level, and income category. Region fixed effects ($\:{\lambda\:}_{r}$) absorb time-invariant regional characteristics, while year fixed effects ($\:{\delta\:}_{t}$) control for common shocks affecting all regions.

The treatment region is defined as TRB2 (Van, Muş, Bitlis, and Hakkari). The primary comparison group consists of geographically proximate NUTS-2 regions, TRA1, TRA2, and TRB1, that were not directly affected by the earthquake. This selection reflects similarities in pre-earthquake socioeconomic characteristics and limits structural differences between treated and comparison regions. These regions share similar baseline socio-demographic structure and pre-earthquake levels/trends in mental health service utilization with the treated region (TRB2), while remaining outside the primary earthquake impact zone. We did not include the southern adjacent regions (Gaziantep/Şanlıurfa/Mardin; TRC1, TRC2, TRC3) in the baseline specification because they were exposed to distinct contemporaneous shocks during the post-2011 period, most notably the Syria conflict-related refugee inflows and associated service demand pressures, raising concerns about control-group contamination. These provinces have faced large-scale Syrian refugee inflows, which academic and policy research shows have placed additional demands on local healthcare systems and altered health service utilization patterns [40–42].

We adopt a dynamic DiD specification that allows treatment effects to vary flexibly over time. This approach facilitates an assessment of pre-treatment trends and the temporal evolution of post-earthquake differences in service utilization. Parallel trends are evaluated through pre-treatment interaction terms and event-study estimates. An alternative parameterization of the model is presented in Appendix C.

To further examine the timing of post-earthquake changes, we estimate specifications that group post-event survey waves into biennial intervals. In addition, we estimate a simplified two-period DiD model using a narrow window around the earthquake (two years before and two years after the event). These specifications are reported in Appendix E and serve to assess sensitivity to the choice of time horizon.

We also evaluate robustness to alternative definitions of the comparison group by estimating specifications that include all other NUTS-2 regions in Türkiye as controls. The corresponding results are reported in Appendix B.

Household income is included in the baseline specifications to account for socioeconomic differences across individuals and regions. Because income may itself respond to disaster exposure, we additionally estimate models excluding income as a robustness check (Appendix H).

### Findings and Results

**Fig. 2:**
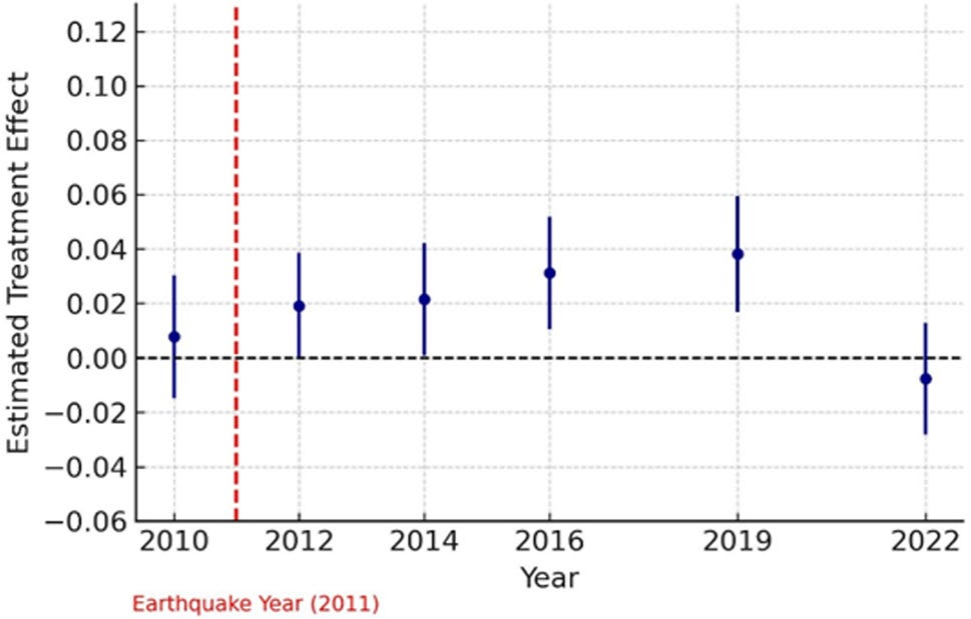
Mental health service use DID treatment effect. Source: Türkiye Health Survey, 2008–2022

Figure 2; Table 1 present the baseline difference-in-differences estimates of changes in mental health service utilization following the 2011 Van earthquake. All specifications include region and year fixed effects, and the full model additionally controls for gender, marital status, age group, education level, and income category. The full regression results, including all control coefficients and year fixed effects, are reported in Appendix Table 3.

**Table 1:** Effects of earthquake on mental health service use following the difference-in-differences model

Variables	No Controls	With Controls
year2010 × treatment	0.008	0.007
	(0.0115)	(0.0115)
year2012 × treatment	0.019*	0.018*
	(0.0100)	(0.0101)
year2014 × treatment	0.021**	0.020*
	(0.0105)	(0.0105)
year2016 × treatment	0.031***	0.029***
	(0.0105)	(0.0105)
year2019 × treatment	0.038***	0.036***
	(0.0109)	(0.0109)
year2022 × treatment	–0.007	–0.009
	(0.0105)	(0.0106)

Source: Türkiye Health Survey, 2008–2022

Notes: All regressions include year and region fixed effects. The full specification additionally controls for gender, marital status, age (teen, adult, middle-aged, senior), education (less than high school, high school, university), and income (low, mid, high, and missing). Standard errors in parentheses. * *p* < 0.1, ** *p* < 0.05, *** *p* < 0.01

To assess the validity of the parallel trends assumption, we conduct a formal pre-trend test by jointly testing the coefficients on treatment interactions in the pre-earthquake period (2008, 2010). The null hypothesis that these coefficients are jointly equal to zero cannot be rejected (F = 1.17, *p* = 0.31), providing no evidence of differential pre-treatment trends between the treatment and comparison regions. Event-study estimates illustrating pre- and post-earthquake dynamics are reported in Fig. 2, with joint pre-trend tests presented in Appendix D. For completeness, an alternative presentation of the event-study estimates is provided in Appendix F. The corresponding event-study specification with full dynamic coefficients is presented in Appendix Fig. 5.

Table 1 reports the estimated treatment effects by survey year. The interaction terms are small and statistically indistinguishable from zero prior to the earthquake, while positive differences emerge in the post-earthquake period. The first post-earthquake estimate in 2012 (*β* = 0.018) corresponds to a 53.7% increase relative to the treatment region’s baseline mental health service utilization rate of 3.35%. The magnitude of the estimated effects increases through the 2016 and 2019 survey waves before attenuating in 2022.

Although several post-earthquake coefficients are statistically significant under conventional clustered standard errors, we additionally report wild-cluster bootstrap *p*-values to account for the limited number of clusters. Under this more conservative inference approach, the estimated effects for 2012, 2016, and 2019 remain statistically supported, while significance weakens in other years (see Appendix Table 2). The pre-treatment findings supporting the parallel trends assumption are also robust to wild-cluster inference.

The estimated treatment effects are further robust to alternative definitions of the comparison group. When all other NUTS-2 regions in Türkiye are used as controls, the magnitude and timing of the post-earthquake estimates remain qualitatively similar, particularly from 2014 onward (Appendix Table 7). Taken together, these results indicate an economically meaningful increase in mental health service utilization emerging several years after the earthquake. Importantly, this delayed pattern should not be interpreted as evidence of a specific causal lag structure. Rather, it reflects the timing of observed utilization differences across survey waves and may be influenced by a combination of behavioral, institutional, and system-wide factors.

**Fig. 3:**
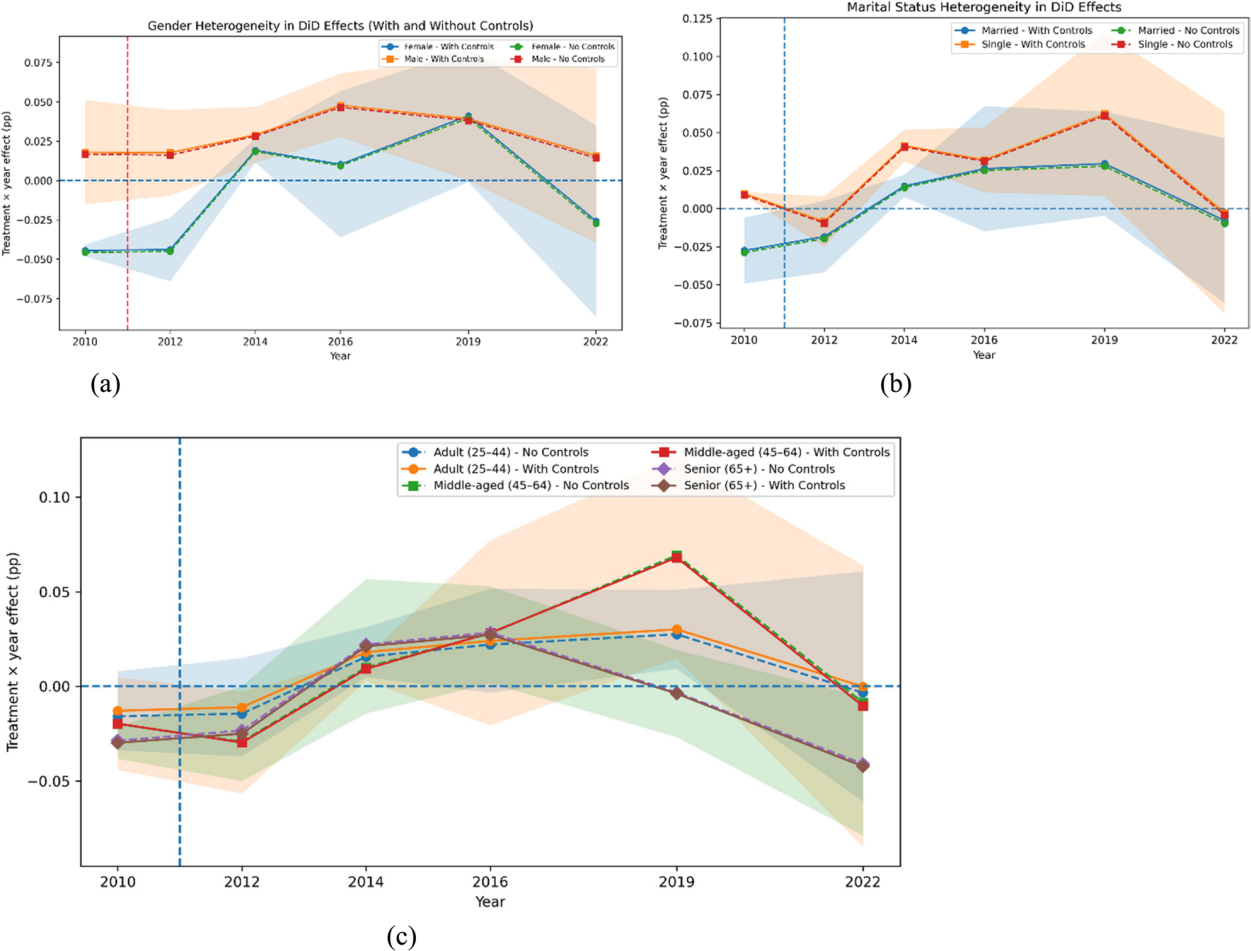
Heterogeneity graphs. Source: Türkiye Health Survey Microdata, 2008–2022. Notes: The year 2008 is the omitted reference period in the event-study specification. Coefficients for 2010, therefore, capture deviations relative to 2008 and serve as placebo leads for assessing pre-treatment dynamics. Regression tables and joint pre-trend tests confirm the absence of differential pre-treatment effects

We next examine whether post-earthquake changes in mental health service utilization vary across demographic groups. Figure 3 presents heterogeneity analyses by gender, marital status, and age group. All specifications include region and year fixed effects and are estimated both with and without individual-level controls. Consistent with the baseline results, no statistically significant differences are observed between treatment and control regions in the pre-treatment period. Post-earthquake differences emerge only after 2014, suggesting that the observed increases in utilization reflect post-disaster dynamics rather than pre-existing trends.

To shed light on potential channels underlying these patterns, we focus on heterogeneity along demographic dimensions that prior research has linked to differential vulnerability and help-seeking behavior following large-scale shocks. The interpretation of these subgroup patterns is discussed in detail in the following section.

While several post-earthquake coefficients are statistically significant under conventional cluster-robust inference, statistical significance weakens when applying wild cluster bootstrap *p*-values, reflecting limited power due to the small number of clusters (*N* = 4). Importantly, the point estimates remain economically meaningful and display a consistent dynamic pattern, with effects emerging after 2014, peaking in the medium run, and attenuating by 2022. We therefore interpret these findings as suggestive evidence of delayed post-disaster behavioral responses rather than definitive causal effects. (See Appendix D Table 11).

Figure 3 presents heterogeneity in post-earthquake mental health service utilization by gender, marital status, and age group. In all specifications, year and region fixed effects are included, and models are estimated both with and without individual-level controls. The year 2008 is the omitted reference period in the event-study specification; coefficients for 2010 thus capture deviations relative to 2008. Regression tables and joint pre-trend tests confirm the absence of differential pre-treatment trends (see Appendix Table 8 for full-sample gender results).

#### Gender

These subgroup differences should be interpreted descriptively and do not represent causal mechanisms. Figure 3(a) illustrates gender-specific dynamics in post-earthquake mental health service utilization. The corresponding regression estimates are reported in Appendix Table 4. While point estimates suggest increases for both men and women in the affected region relative to controls, the confidence intervals indicate substantial uncertainty, and gender differences are not consistently statistically distinguishable across survey waves. The estimated effects for men tend to be larger in magnitude in the mid- to long-run (particularly around 2016–2019), whereas coefficients for women are generally smaller and more imprecisely estimated.

This pattern contrasts with national evidence from Türkiye, where women are more likely than men to report poor mental health and to use mental health services [26]. A plausible explanation lies in differential access rather than differential need. Prior research shows that women in eastern Türkiye face greater barriers to formal mental health care, including social stigma, restricted mobility, household responsibilities, and family pressure [27, 28]. Consistent with this interpretation, population-level evidence documents higher rates of cost-related unmet health care needs among women than men in Türkiye [29]. Taken together, these findings suggest that the observed gender differences in utilization reflect unequal access and help-seeking conditions rather than lower underlying mental health needs among women.

#### Marital status

These subgroup differences should be interpreted descriptively and do not represent causal mechanisms. Figure 3(b) illustrates heterogeneity in post-earthquake mental health service utilization by marital status. The corresponding regression estimates are reported in Appendix Table 5. Point estimates suggest increases in utilization for both married and single individuals in the affected region relative to controls. While estimates for single respondents tend to be larger in magnitude in some post-earthquake survey waves, the confidence intervals indicate substantial overlap between groups, and differences are not consistently statistically distinguishable over time. Overall, the graphical evidence points to modest and time-varying differences by marital status rather than a persistent divergence. This pattern is consistent with the notion that spousal support may shape help-seeking behavior following large shocks, but the results do not support a strong or uniform marital-status gradient in mental health service use. Using the full Türkiye sample as the control group yields qualitatively similar marital-status patterns (Appendix Table 9).

#### Age groups

These subgroup differences should be interpreted descriptively and do not represent causal mechanisms. Figure 3(c) presents heterogeneity in post-earthquake mental health service utilization across age groups. The corresponding regression estimates are reported in Appendix Table 6. The estimates reveal heterogeneous and time-varying patterns rather than a clear age gradient. Seniors exhibit positive differences relative to control regions in some early post-earthquake years, but these effects are not persistent over time. For adults and middle-aged individuals, post-earthquake coefficients fluctuate around zero and are generally estimated with considerable uncertainty, providing limited evidence of sustained increases in utilization.

Appendix Table 10 reports age-specific difference-in-differences estimates using the full Türkiye sample as the control group, with separate regressions for teens, adults, middle-aged adults, and seniors. Consistent with the event-study evidence, treatment–year interactions are small and statistically insignificant in the pre-earthquake period (2010–2012) across all age groups, supporting the parallel trends assumption. In the post-earthquake period, the estimates do not display a uniform or monotonic age pattern. While seniors show statistically significant increases in utilization in the early post-earthquake years, these effects attenuate in later waves. For working-age and middle-aged individuals, post-2014 coefficients are generally modest in magnitude and statistically imprecise.

Taken together, the age-heterogeneity results suggest that the aggregate post-earthquake increase in mental health service utilization documented in the main analysis is not driven by a single age group but reflects modest and heterogeneous responses across cohorts. Given the repeated cross-sectional nature of the Türkiye Health Survey and the transition of individuals across age categories over time, these estimates should be interpreted as contemporaneous differences across age groups rather than life-cycle trajectories. Earlier versions of the analysis grouped individuals into three broad age categories; to assess sensitivity to alternative age definitions, we re-estimate the age heterogeneity using a finer four-group classification, reported in Appendix Table 10.

### Robustness and additional outcomes

To further assess robustness, we conduct several additional analyses. A biennial specification confirms the absence of effects in the immediate post-earthquake period (2012–2014), serving as a near-term placebo test (Appendix Table 12). We also estimate placebo-region specifications assigning treatment status to Erzurum (TRA1), which yield no pre-treatment differences and post-2011 patterns opposite in sign to the main estimates (Appendix Table 15. In addition, re-estimating all models excluding income produces qualitatively similar results (Appendix H).

As a complementary outcome, we examine self-rated health (SRH), coded such that higher values indicate poorer perceived health. Dynamic difference-in-differences estimates show improvements in self-rated health in the affected region relative to control regions in the post-earthquake period, with the largest changes observed in 2019 and partial attenuation by 2022 (Appendix Table 13). This pattern is broadly consistent with the utilization results.

### Interpretation and limitations

Several subgroup patterns are discussed through mechanisms such as stigma, access barriers, and reliance on informal support networks, which are well documented in the disaster and mental health literature. However, the Türkiye Health Survey does not contain direct measures of service availability, facility proximity, or household support structures. Accordingly, these mechanisms should be viewed as interpretative frameworks rather than empirically tested channels. While the difference-in-differences design strengthens causal interpretation relative to descriptive comparisons, the results are best interpreted as changes in observed mental health service utilization associated with earthquake exposure rather than definitive causal effects.

## Discussion

Our findings indicate a sustained increase in mental health service utilization following the 2011 Van earthquake, with effects peaking several years after the shock and attenuating by 2022. The decline observed in 2022 likely reflects the disruptive effects of the COVID-19 pandemic, including temporary closures of outpatient services, reduced access to non-emergency care, and widespread pandemic-related stress, which may have compressed regional differences in service use.

The delayed and demographically differentiated nature of the response is consistent with insights from behavioral economics and health service use theory [33, 34]. Mental health care is particularly prone to postponement because its perceived benefits are often delayed, while its costs—such as stigma, time, and emotional discomfort—are immediate. As a result, individuals exposed to traumatic events may initially defer seeking care, prioritizing more urgent material needs or expecting symptoms to resolve on their own. Over time, as distress persists or interferes with daily functioning, perceived need for professional care increases, leading to delayed utilization. This distinction between latent psychological distress and perceived need is a core element of the Andersen Behavioral Model of Health Services Use [35]. These behavioral interpretations should be viewed as plausible frameworks rather than empirically identified mechanisms.

Heterogeneity analyses further underscore the socially embedded nature of post-disaster health-seeking behavior. Lower utilization among women in the affected region likely reflects structural constraints rather than lower underlying need. Prior research documents that women in eastern Türkiye face stigma, mobility limitations, caregiving responsibilities, and family pressures that suppress formal service use even in the presence of psychological distress [27, 28]. Consistent with this interpretation, population-level evidence shows higher rates of cost-related unmet healthcare needs among women than men in Türkiye [29]. In contrast, higher and more persistent utilization among men may reflect the absence of informal coping outlets and gender norms discouraging emotional disclosure, leading to delayed but pronounced engagement with formal mental health services once symptoms accumulate [26].

Similarly, larger and earlier effects among single individuals may be linked to weaker informal support networks and greater autonomy in health-related decision-making, consistent with vulnerability theory [11]. Age-specific patterns indicate that working-age and middle-aged adults experience the most persistent increases in utilization, while effects among seniors remain limited. Because the Türkiye Health Survey consists of repeated cross-sections rather than a panel, these age-specific estimates should be interpreted as differences across age groups at each survey wave rather than life-cycle trajectories for the same individuals.

Supply-side explanations cannot be fully ruled out. Contextual evidence from Health Statistics Yearbooks published by the Turkish Ministry of Health [30–32] suggests that healthcare capacity in Eastern Anatolia expanded gradually during the 2010s, but there is no indication of an abrupt, region-specific increase in mental health service supply following the earthquake. Given the aggregate nature of these statistics and their mismatch with the NUTS-2 level used in our analysis, supply-side developments are best viewed as background conditions rather than direct drivers of the observed patterns. More broadly, improvements in insurance coverage, reductions in financial barriers, increased screening in primary care, and gradual strengthening of referral pathways may have interacted with evolving demand to shape utilization over time. An important concern is whether the observed post-earthquake differences reflect the effects of the Van earthquake itself or broader national trends in mental health service utilization. During the study period, Türkiye experienced system-wide changes, including gradual expansion of mental health services, increased awareness, and evolving referral practices. These concurrent developments cannot be fully ruled out. Accordingly, the estimates are best interpreted as regional differences in observed utilization emerging in the post-earthquake period, rather than as effects that isolate the earthquake from all other contemporaneous trends.If the observed pattern merely reflected nationwide secular increases, similar divergence would plausibly be observed across other eastern regions exposed to comparable macroeconomic conditions. In contrast, the estimated divergence appears more pronounced in the earthquake-affected region relative to the proximate control regions included in the empirical design.

An important concern is whether the delayed increase in utilization can be attributed to the earthquake in a context characterized by multiple concurrent shocks, including macroeconomic volatility, political instability, migration flows, and regional conflict dynamics. While the Türkiye Health Survey does not allow us to directly measure exposure to these stressors, several features of the empirical patterns support a disaster-related interpretation. The timing of the estimated effects aligns with the post-earthquake period rather than earlier regional shocks, and the strongest increases are concentrated in the earthquake-affected region relative to other eastern regions exposed to similar broader conditions.

Several limitations warrant consideration. Treatment is defined at the NUTS-2 level rather than by distance to the earthquake epicenter, which may introduce within-region measurement error and precludes explicit modeling of spillovers. Because treatment is defined at the NUTS-2 level for both treated and control regions, any within-region measurement error in exposure is likely to attenuate estimated differences toward zero rather than generate spurious treatment effects. In addition, the data do not allow us to disentangle demand-side behavioral responses from supply-side capacity expansion or to directly observe mechanisms such as facility proximity, transportation constraints, or household support structures. Accordingly, the estimated effects should be interpreted as changes in observed mental health service utilization associated with earthquake exposure rather than definitive causal effects.

Despite these limitations, the results are robust to alternative control groups, exclusion of income, placebo-region tests, and complementary analyses using self-rated health. Given the small number of clusters, statistical power is limited; accordingly, we emphasize the consistency and economic magnitude of the dynamic pattern rather than isolated significance levels. Taken together, the findings highlight the long-term mental health burden of natural disasters and the importance of sustained psychological care in recovery planning. By combining a quasi-experimental design with rich subgroup analysis, this study contributes new evidence from an upper-middle-income country context and provides a replicable framework for analyzing post-disaster health-seeking behavior in settings where stigma, access barriers, and institutional capacity play a central role [36, 37].

## Conclusion

This study examines how exposure to a major natural disaster translates into changes in formal mental health service utilization over time. Using repeated cross-sectional data from the Türkiye Health Survey and a dynamic difference-in-differences design, we document a sustained increase in mental health service use following the 2011 Van earthquake. Importantly, this increase does not emerge immediately after the shock but develops gradually, peaks several years later, and attenuates by 2022. These patterns are robust across alternative control groups, placebo tests, and complementary outcomes, and should be interpreted as changes in observed utilization associated with disaster exposure rather than definitive causal effects.

The findings contribute to the disaster and mental health literature in several ways. First, they shift attention from short-run psychological distress to the medium- and long-run use of formal mental health services, capturing behavioral responses that unfold gradually after large shocks. Second, by focusing on Türkiye, an upper-middle-income country with an expanding but uneven mental health infrastructure, the study provides evidence from a context that remains underrepresented in quasi-experimental disaster research. Third, the delayed trajectory of service use highlights the importance of behavioral mechanisms, such as stigma, delayed recognition of need, and access barriers, in shaping post-disaster help-seeking behavior. From a public health perspective, these results underscore that mental health needs following disasters may persist long after physical reconstruction is completed, calling for sustained and adaptive recovery planning.

Several limitations should be acknowledged. Treatment is defined at the regional (NUTS-2) level, preventing distance-based exposure measures and explicit modeling of spillovers. The data do not allow us to disentangle demand-side behavioral responses from supply-side expansions in mental health services, nor to directly observe mechanisms such as facility proximity or household support structures. Future research combining richer administrative data, geocoded information, or alternative identification strategies, such as synthetic control methods, could further refine our understanding of the long-term mental health consequences of disasters. Despite these limitations, the present study provides credible and policy-relevant evidence on how large-scale shocks shape mental health service utilization over time in middle-income settings.

## Figures and Tables

**Fig. 4:**
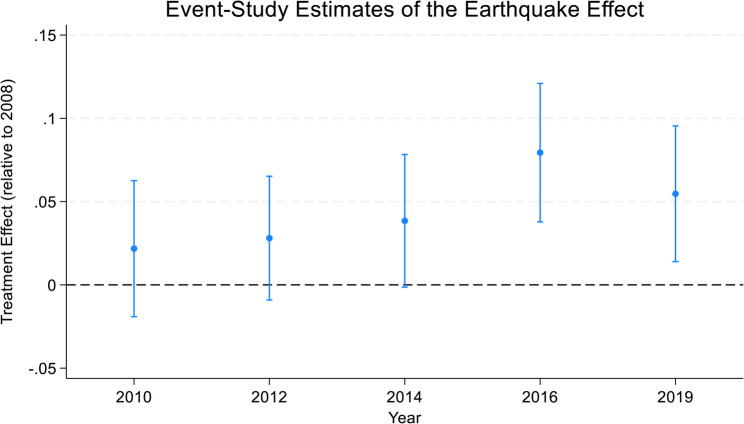
Event-study estimates of the impact of the 2011 earthquake on psychological service utilization. Source: Türkiye Health Survey Microdata (2008–2022) made available by the Turkish Statistical Institute (TUIK) under a special access agreement. For further details, see https://www.tuik.gov.tr/. Notes: Coefficients represent year-by-treatment interactions from a difference-in-differences model normalized to zero in 2008. The vertical dashed line marks the 2011 earthquake. Models include regional and year-fixed effects, as well as individual-level controls. 95% confidence intervals are shown

**Fig. 5:**
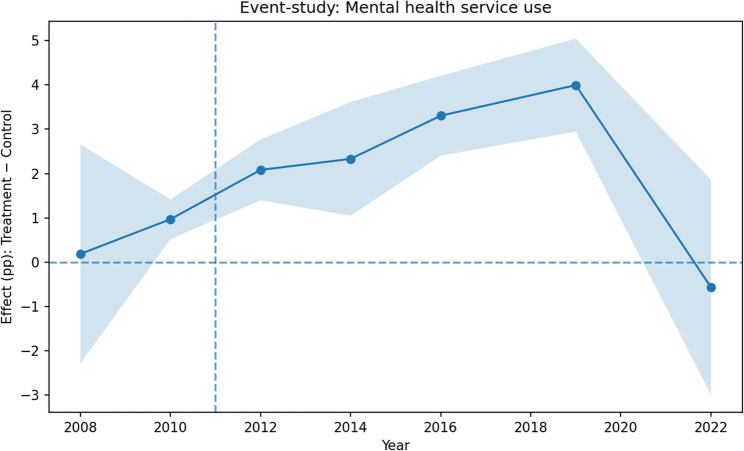
Event-study difference-in-differences estimates of mental health service use (percentage points). Source: Türkiye Health Survey Microdata (2008–2022) made available by the Turkish Statistical Institute (TUIK) under a special access agreement. For further details, see https://www.tuik.gov.tr/. Notes: Points indicate treatment×year coefficients; shaded bands are 95% confidence intervals. The dashed vertical line marks the 2011 Van earthquake; the horizontal line denotes zero. Models include region (NUTS-2) and year fixed effects. Given four clusters, wild-cluster bootstrap *p*-values are reported in Appendix Table 11

## Data Availability

The Türkiye Health Survey microdata that support the findings of this study are available from the Turkish Statistical Institute (TÜİK), but restrictions apply to their availability. Data were used under license for the current study and are not publicly available. Data can be requested directly from TÜİK via their official website: [**https://www.tuik.gov.tr/**](https:/www.tuik.gov.tr) . Researchers can apply for microdata access through the relevant section on the TÜİK website.

## Appendix

### Appendix A

**Table 2:** Summary statistics

Variable	Treatment		Control		
	Before	After	Before	After	
Mental health service use	0.0335 (0.0041)	0.0540 (0.0027)	0.0266 (0.0032)	0.0308 (0.0017)	
Female	0.5301 (0.0112)	0.5338 (0.0060)	0.5859 (0.0098)	0.5448 (0.0050)	
Married	0.6687 (0.0106)	0.6234 (0.0059)	0.7005 (0.0091)	0.6068 (0.0049)	
Mid-income	0.4335 (0.0111)	0.2888 (0.0055)	0.2951 (0.0091)	0.2204 (0.0042)	
High-income	0.1533 (0.0081)	0.1665 (0.0045)	0.0881 (0.0056)	0.0873 (0.0028)	
Income missing	0.0010 (0.0007)	0.3006 (0.0055)	0.0146 (0.0024)	0.3218 (0.0047)	
Adult (25–34)	0.3778 (0.0109)	0.3486 (0.0058)	0.3987 (0.0097)	0.3295 (0.0047)	
Middle adult (35–64)	0.3060 (0.0104)	0.3085 (0.0056)	0.2793 (0.0089)	0.2553 (0.0044)	
Senior (65+)	0.1416 (0.0078)	0.1390 (0.0042)	0.1217 (0.0065)	0.1228 (0.0033)	
High school	0.1482 (0.0080)	0.1908 (0.0048)	0.0897 (0.0057)	0.1213 (0.0033)	
University	0.0971 (0.0067)	0.1791 (0.0046)	0.1217 (0.0065)	0.0877 (0.0029)	
N	1,977	6,834	2,531	9,827	21,169

Source: Türkiye Health Survey Microdata (2008–2022) made available by the Turkish Statistical Institute (TUIK) under a special access agreement. For further details, see https://www.tuik.gov.tr/ (Standard errors in the parentheses)

**Table 3:** Effects of earthquake on mental health service use following the difference-in-differences model

Variables	No Controls	With Controls	
Panel A: Main Results			
year2010 × treatment	0.008	0.007	
	(0.0115)	(0.0115)	
year2012 × treatment	0.019**	0.018*	
	(0.0100)	(0.0101)	
year2014 × treatment	0.021**	0.020**	
	(0.0105)	(0.0105)	
year2016 × treatment	0.031***	0.029***	
	(0.0105)	(0.0105)	
year2019 × treatment	0.038***	0.036***	
	(0.0109)	(0.0109)	
year2022 × treatment	–0.007	–0.009	
	(0.0105)	(0.0106)	
year2010	–0.010	–0.010	
	(0.0076)	(0.0076)	
year2012	–0.010	–0.010	
	(0.0068)	(0.0068)	
year2014	0.001	0.004	
	(0.0070)	(0.0070)	
year2016	0.001	0.003	
	(0.0070)	(0.0070)	
year2019	0.003	0.007	
	(0.0072)	(0.0093)	
year2022	0.008	0.010	
	(0.0069)	(0.0090)	
Panel B: Control Variables			**N**
female		0.021***	21,169
		(0.0027)	
married		–0.019***	21,169
		(0.0036)	
adult		0.033***	21,169
		(0.0044)	
middle_adult		0.038***	21,169
		(0.0047)	
senior		0.024***	21,169
		(0.0050)	
mid_income		0.004	21,169
		(0.0035)	
high_income		–0.004	21,169
		(0.0048)	
income_miss		–0.001	21,169
		(0.0071)	
high_school		0.002	21,169
		(0.0040)	
university		–0.003	21,169
		(0.0044)	
N (Total)			21,142

Source: Türkiye Health Survey Microdata (2008–2022) made available by the Turkish Statistical Institute (TUIK) under a special access agreement. For further details, see https://www.tuik.gov.tr/ (Standard errors in the parentheses)

Notes: **p* < 0.10, ***p* < 0.05, ****p* < 0.01. All regression includes year and region fixed effects. (Standard errors in the parentheses) The 2010 coefficient is not statistically significant in any specification, consistent with the parallel trends assumption

**Table 4:** Gender heterogeneity

Variable	Female No Controls	Female With Controls	Male No Controls	Male With Controls
treatment × 2010	0.0015	0.0020	0.0106	0.0102
	(0.0172)	(0.0172)	(0.0145)	(0.0145)
treatment × 2012	0.0148	0.0158	0.0193*	0.0197*
	(0.0152)	(0.0152)	(0.0126)	(0.0126)
treatment × 2014	0.0182	0.0172	0.0238**	0.0239**
	(0.0160)	(0.0160)	(0.0130)	(0.0130)
treatment × 2016	0.0183	0.0165	0.0442***	0.0440***
	(0.0160)	(0.0160)	(0.0131)	(0.0131)
treatment × 2019	0.0342**	0.0340**	0.0414***	0.0411***
	(0.0166)	(0.0166)	(0.0137)	(0.0137)
treatment × 2022	–0.0199	–0.0194	0.0050	0.0030
	(0.0161)	(0.0161)	(0.0130)	(0.0130)
N	11515	11515	9627	9627

Source: Türkiye Health Survey Microdata (2008–2022) made available by the Turkish Statistical Institute (TUIK) under a special access agreement. For further details, see https://www.tuik.gov.tr/ (Standard errors in the parentheses)

Notes: All regressions include year and NUTS2 region fixed effects. The full specification additionally controls for gender, marital status, age groupings (teen, adult, middle-adut, senior), education (less than high school, high school, university), and income indicators (low, mid, high, and missing). Standard errors in parentheses. Statistical significance: **p* < 0.10, ***p* < 0.05, ****p* < 0.01. Although the female sample size is slightly larger than that of males, the estimated treatment effects for women are not statistically significant in most years. This may reflect the social and cultural context of the eastern region, where women’s access to mental health services could be constrained by factors such as stigma, household roles, and limited autonomy in seeking care. As a result, changes in utilization behavior might be less observable among women, even if underlying needs exist. These patterns suggest the importance of considering gendered access barriers when interpreting heterogeneous effects

**Table 5:** Marital status heterogeneity

Variable	Married No Controls	Married With Controls	Single No Controls	Single With Controls
treatment × 2010	0.0086	0.0087	0.0057	0.0060
	(0.0139)	(0.0139)	(0.0204)	(0.0204)
treatment × 2012	0.0130	0.0136	0.0316 **	0.0273*
	(0.0121)	(0.0121)	(0.0180)	(0.0180)
treatment × 2014	0.0145	0.0149	0.0335 **	0.0316 **
	(0.0131)	(0.0131)	(0.0178)	(0.0178)
treatment × 2016	0.0305 **	0.0323 **	0.0371 **	0.0266*
	(0.0133)	(0.0133)	(0.0177)	(0.0177)
treatment × 2019	0.0309 **	0.0336 ***	0.0573 ***	0.0431 **
	(0.0137)	(0.0137)	(0.0183)	(0.0183)
treatment × 2022	–0.0156	–0.0143	–0.0068	0.0007
	(0.0129)	(0.0129)	(0.0184)	(0.0184)

Source: Türkiye Health Survey Microdata (2008–2022) made available by the Turkish Statistical Institute (TUIK) under a special access agreement. For further details, see https://www.tuik.gov.tr/ (Standard errors in the parentheses)

Notes: All regressions include year and region fixed effects. The full specification additionally controls for gender, marital status, age groupings (teen, adult, middle-adult, senior), education (less than high school, high school, university), and income indicators (low, mid, high, and missing).*Standard errors in parentheses. Statistical significance: **p* < 0.10, ***p* < 0.05, ****p* < 0.01

**Table 6:** Age heterogeneity

Variable	Adult No Controls	Adult With Controls	Middle Adult No Controls	Middle Adult With Controls	Senior No Controls	Senior With Controls
treatment × 2010	0.0178	0.0202	0.0039	0.0035	–0.0328	–0.0338
	(0.0193)	(0.0193)	(0.0235)	(0.0235)	(0.0322)	(0.0322)
treatment × 2012	0.0366 **	0.0411 **	0.0153	0.0115	–0.0307	–0.0309
	(0.0166)	(0.0166)	(0.0205)	(0.0205)	(0.0293)	(0.0293)
treatment × 2014	0.0156	0.0179	0.0141	0.0094	0.0021	–0.0002
	(0.0183)	(0.0183)	(0.0220)	(0.0220)	(0.0312)	(0.0312)
treatment × 2016	0.0254*	0.0291*	0.0350*	0.0337	0.0177	0.0159
	(0.0183)	(0.0183)	(0.0223)	(0.0223)	(0.0317)	(0.0317)
treatment × 2019	0.0214***	0.0232***	0.0747	0.0732	0.0105	0.0099
	(0.0193)	(0.0193)	(0.0227)	(0.0227)	(0.0315)	(0.0315)
treatment × 2022	–0.0071	–0.0047	–0.0058	–0.0107	–0.0579 *	–0.0606 **
	(0.0175)	(0.0175)	(0.0184)	(0.0184)	(0.0302)	(0.0302)

Source: Türkiye Health Survey Microdata (2008–2022) made available by the Turkish Statistical Institute (TUIK) under a special access agreement. For further details, see https://www.tuik.gov.tr/ (Standard errors in the parentheses)

Notes: All regressions include year and region fixed effects. The full specification additionally controls for gender, marital status, age groupings (teen, adult, middle-aged, senior), education (less than high school, high school, university), and income indicators (low, mid, high, and missing). Standard errors in parentheses. Statistical significance: **p* < 0.10, ***p* < 0.05, ****p* < 0.01 The 2010 coefficient is not statistically significant in any specification, consistent with the parallel trends assumption

### Appendix B Full sample

**Table 7:** DiD – Full Sample (With Controls)

Variables	With Controls
Panel A: Main Results	
year2010 × treatment	0.0164*
	(0.0095)
year2012 × treatment	0.0061
	(0.0081)
year2014 × treatment	0.0252***
	(0.0088)
year2016 × treatment	0.0440***
	(0.0090)
year2019 × treatment	0.0370***
	(0.0092)
Panel B: Control Variables	
female	0.0129***
	(0.0013)
married	−0.0062***
	(0.0014)
high_school	−0.0003
	(0.0019)
university	0.0027
	(0.0019)
adult	0.0093***
	(0.0018)
middle_adult	0.0050**
	(0.0019)
senior	0.0019
	(0.0020)
Region FE (IBBS2)	Yes
Year FE	Yes
Observations (N)	118,610

Source: Türkiye Health Survey Microdata (2008–2022) made available by the Turkish Statistical Institute (TUIK) under a special access agreement. For further details, see https://www.tuik.gov.tr/ (Standard errors in the parentheses)

Notes: All regressions include year and region fixed effects. The full specification additionally controls for gender, marital status, age groupings (teen, adult, middle-aged, senior), education (less than high school, high school, university), and income indicators (low, mid, high, and missing). Standard errors in parentheses. Statistical significance: **p* < 0.10, ***p* < 0.05, ****p* < 0.01 The 2010 coefficient is not statistically significant in any specification, consistent with the parallel trends assumption

**Table 8:** Gender heterogeneity results using the full Türkiye sample as the control group

Variables	Male	Female
year2010 × treatment	0.0271**	0.0078
	(0.0117)	(0.0144)
year2012 × treatment	0.0153	−0.0005
	(0.0099)	(0.0124)
year2014 × treatment	0.0212**	0.0292**
	(0.0107)	(0.0136)
year2016 × treatment	0.0525***	0.0368***
	(0.0109)	(0.0137)
year2019 × treatment	0.0288**	0.0431***
	(0.0113)	(0.0141)
Region FE (IBBS2)	Yes	Yes
Year FE	Yes	Yes
Observations (N)	54,325	64,285

Source: Türkiye Health Survey Microdata (2008–2022) made available by the Turkish Statistical Institute (TUIK) under a special access agreement. For further details, see https://www.tuik.gov.tr/ (Standard errors in the parentheses)

Notes: All regressions include year and region fixed effects. The full specification additionally controls for marital status, age groupings (teen, adult, middle-adult, senior), education (less than high school, high school, university), and income indicators (low, mid, high, and missing). Standard errors in parentheses. Statistical significance: * *p* < 0.10, ** *p* < 0.05, *** *p* < 0.01

**Table 9:** Marital Status heterogeneity results using the full Türkiye sample as the control group

Variables	Not married	Married
year2010 × treatment	0.0127	0.0199*
	(0.0172)	(0.0113)
year2012 × treatment	−0.0049	0.0124
	(0.0148)	(0.0097)
year2014 × treatment	0.0309*	0.0237**
	(0.0161)	(0.0105)
year2016 × treatment	0.0224	0.0552***
	(0.0166)	(0.0106)
year2019 × treatment	0.0442***	0.0350***
	(0.0168)	(0.0110)
Region FE (IBBS2)	Yes	Yes
Year FE	Yes	Yes
Observations (N)	37,573	81,037

Source: Türkiye Health Survey Microdata (2008–2022) made available by the Turkish Statistical Institute (TUIK) under a special access agreement. For further details, see https://www.tuik.gov.tr/ (Standard errors in the parentheses)

Notes: All regressions include year and region fixed effects. The full specification additionally controls for marital status, age groupings (teen, adult, middle-aged, senior), education (less than high school, high school, university), and income indicators (low, mid, high, and missing). Standard errors in parentheses. Statistical significance: * *p* < 0.10, ** *p* < 0.05, *** *p* < 0.01

**Table 10:** Age heterogeneity heterogeneity results using the full Türkiye sample as the control group

Variables	Teen	Adult	Middle adult	Senior
2010 × Treatment	0.0058	0.0066	0.0076	0.0221***
	(0.0189)	(0.0164)	(0.0176)	(0.0078)
2012 × Treatment	0.0007	−0.0009	0.0063	0.0177***
	(0.0164)	(0.0119)	(0.0113)	(0.0059)
2014 × Treatment	0.0216*	−0.0058	−0.0164**	0.0074
	(0.0120)	(0.0079)	(0.0066)	(0.0112)
2016 × Treatment	−0.0006	−0.0035	0.0154*	−0.0060
	(0.0104)	(0.0078)	(0.0084)	(0.0092)
2019 × Treatment	0.0133	−0.0003	−0.0143	0.0088
	(0.0086)	(0.0113)	(0.0105)	(0.0110)
2022 × Treatment	−0.0101	−0.0152**	−0.0042	0.0095
	(0.0109)	(0.0060)	(0.0103)	(0.0097)
Region FE (IBBS2)	Yes	Yes	Yes	Yes
Year FE	Yes	Yes	Yes	Yes
Observations (N)	22,251	48,946	37,689	16,019

Source: Türkiye Health Survey Microdata (2008–2022) made available by the Turkish Statistical Institute (TUIK) under a special access agreement. For further details, see https://www.tuik.gov.tr/ (Standard errors in the parentheses)

Notes: All regressions include year and region fixed effects. The full specification additionally controls for gender, marital status, age groupings (teen, adult, middle-adult, senior), education (less than high school, high school, university), and income indicators (low, mid, high, and missing). Standard errors are reported in parentheses. Statistical significance: * *p* < 0.10, ** *p* < 0.05, *** *p* < 0.01

### Appendix C: Formal tests of pre-trends and alternative normalizations

This appendix is provided solely for transparency and does not introduce a separate empirical model beyond the dynamic difference-in-differences specification presented in the main text. It reports an alternative normalization of the time dimension used to assess pre-treatment trends and post-treatment dynamics. The results are fully consistent with the baseline dynamic DiD estimates and confirm the absence of differential pre-treatment trends between treatment and control regions. As the main specification already captures both parallel trends and post-treatment effects, the results in this appendix are included for completeness rather than interpretation.

Using pre-treatment waves from 2008 to 2010, we assess the parallel trends assumption and show that the treatment and control regions followed similar paths in mental health service utilization prior to the earthquake. We implement an event-study specification normalized to the pre-earthquake period.

1 $$\:{Y\:}_{i,r,t}=\:\alpha\:+\sum\:_{k\ne\:-1}{\theta\:}_{k}\:\left({treatment}_{r}\times\:1\left\{t\:-\:\tau\:=k\right\}\right)+{\lambda\:}_{r}+\:{\delta\:\:}_{t}+\:\gamma\:\:{X}_{i,r,t}\:+\:{\epsilon\:}_{\:i,r,t}$$

In Eq. (1) $\:\tau\:=2011$ denotes the earthquake year, $\:k$ indexes years relative to the event (with $\:k=-1\:$ as the omitted reference period), and the coefficients $\:{\theta\:}_{k}$ trace the dynamic treatment effects over time. Region ($\:{\lambda\:}_{r}$) and year ($\:{\delta\:}_{t}$) fixed effects are included, and $\:{X}_{i,r,t}$ represents individual-level control variables. Given repeated cross-sections, two pre-earthquake waves, four regional clusters, and potential post-disaster changes in supply and population composition (e.g., migration), we interpret our estimates as changes in observed utilization rather than definitive causal effects. We acknowledge that potential post-disaster migration from the Van region may influence the observed utilization trends. However, due to data limitations, our estimates reflect net utilization changes among those surveyed, not adjusted for population compositional shifts.

### Appendix D: Event-study analysis, parallel trends and wild cluster bootstrap estimates

**Table 11:** Wild cluster bootstrap inference for event-study estimates

Year	Effect (pp)	SE (pp)	*p*-value (cluster)	*p*-value (wild)
2010	0.97	0.23	0.000	0.250
2012	2.08	0.35	0.000	0.375
2014	2.33	0.66	0.000	0.000
2016	3.30	0.46	0.000	0.375
2019	3.99	0.53	0.000	0.125
2022	−0.56	1.24	0.648	0.500

Source: Türkiye Health Survey Microdata (2008–2022) made available by the Turkish Statistical Institute (TUIK) under a special access agreement. For further details, see https://www.tuik.gov.tr/

Notes: Reported coefficients correspond to year-by-treatment interaction terms from the event-study difference-in-differences specification, expressed in percentage points relative to the 2008 baseline. Conventional cluster-robust *p*-values are reported alongside wild cluster bootstrap *p*-values, clustered at the NUTS-2 level (ıbbs2_sozde). Given the small number of clusters (four regions), wild bootstrap inference is conservative and relies on the full universe of Rademacher weights (2⁴ = 16), leading to discrete *p*-values. Pre-treatment coefficients are statistically indistinguishable from zero, supporting the parallel trends assumption. Post-earthquake point estimates are positive and largest in the 2016–2019 period, with attenuation by 2022

### Appendix E

**Table 12:** Biennial interaction estimates

Variable (Interaction)	Coefficient	Robust SE	t-stat	*P*-value	Interpretation
postvan×treatment(2012–2014)	−0.021	0.040	−0.53	0.602	Placebo (No immediate effect)
Controls	Included				
Region & Year FE	Included				
Observations	71,279				

Source: Türkiye Health Survey Microdata (2008–2022) made available by the Turkish Statistical Institute (TUIK) under a special access agreement. For further details, see https://www.tuik.gov.tr/

### Appendix F

Event-study estimates show no pre-treatment differences and a sustained post-2011 increase in mental health service use in the treatment region relative to controls, peaking at about 3–4% points in 2016–2019 and attenuating by 2022. A pre-treatment test supports parallel trends (2008 lead: cluster-robust F(1,3) = 0.022, *p* = 0.892; wild-cluster bootstrap *p* = 0.879).

At the peak (2016–2019), the estimated increase of 3–4% points corresponds to roughly 30–40 additional users per 1,000 adults, relative to the 2010 baseline rate in the treatment region. In the full model, we see that women, single and people in adulthood are significantly more likely to see mental health professionals. Although conventional cluster-robust inference suggests statistically significant effects, the small number of clusters (*N* = 4) warrants caution. Wild-cluster bootstrap *p*-values are reported following Cameron, Gelbach, and Miller [43], which provides more conservative yet more reliable inference under a few clusters.

### Appendix G

**Table 13:** Dynamic difference-in-differences estimates of poor self-rated health

Variable	Coefficient
2016	0.054*** (0.013)
2019	0.069*** (0.016)
2022	−0.019 (0.016)
Treatment × Year (DiD effects)	
Treatment × 2016	−0.045** (0.017)
Treatment × 2019	−0.081*** (0.018)
Treatment × 2022	−0.037** (0.016)
Age groups (ref: Teen, 15–24)	
Adult (25–44)	0.079*** (0.010)
Middle-adult (45–64)	0.189*** (0.011)
Senior (65+)	0.333*** (0.012)
Other controls	
Married	−0.046*** (0.008)
Missing income	−0.021 (0.014)
Middle income	−0.056*** (0.010)
High income	−0.076*** (0.011)
High school	−0.045*** (0.010)
University	−0.058*** (0.010)
Constant	0.068*** (0.011)
Region fixed effects (NUTS-2)	Yes
Observations	10,805
R-squared	0.135

Source: Türkiye Health Survey Microdata (2008–2022) made available by the Turkish Statistical Institute (TUIK) under a special access agreement. For further details, see https://www.tuik.gov.tr/ (Standard errors in the parentheses)

Notes: Self-rated health (SRH) is coded such that 1 indicates poor health and 0 indicates good health; therefore, negative coefficients imply improvements in health. The model includes region fixed effects. The treatment indicator is absorbed by region fixed effects. Robust standard errors are reported in parentheses. *** *p* < 0.01, ** *p* < 0.05, * *p* < 0.10

### Appendix H: Robustness checks excluding income

**Table 14:** Robustness checks excluding income

Variables	Coefficient	Std. Error
2010 × Treatment	−0.016	(0.012)
2012 × Treatment	−0.016	(0.010)
2014 × Treatment	0.024**	(0.011)
2016 × Treatment	0.028**	(0.012)
2019 × Treatment	0.040***	(0.012)
2022 × Treatment	−0.006	(0.011)
Year fixed effects	Yes	
Region fixed effects (NUTS-2)	Yes	
Controls		
Married	−0.010***	(0.004)
Adult (25–44)	0.009*	(0.005)
Middle-adult (45–64)	0.013***	(0.005)
Senior (65+)	0.008	(0.006)
High school	−0.006	(0.005)
University	−0.008*	(0.005)
Constant	0.033***	(0.005)
Observations	19,377	

Source: Türkiye Health Survey Microdata (2008–2022) made available by the Turkish Statistical Institute (TUIK) under a special access agreement. For further details, see https://www.tuik.gov.tr/ (Standard errors in the parentheses)

Notes: This table reports robustness checks excluding income from the main specifications to address concerns that income may be affected by disaster exposure. The dependent variable is a binary indicator equal to one if the respondent visited a psychologist or psychiatrist in the past year. All models include year and NUTS-2 region fixed effects. Robust standard errors are reported in parentheses. *** *p* < 0.01, ** *p* < 0.05, * *p* < 0.10

### Appendix I

**Table 15:** Placebo Test

Variables	Coefficient	Std. Error
2010 × Placebo	0.0005	(0.0051)
2012 × Placebo	0.0022	(0.0036)
2014 × Placebo	−0.0234***	(0.0029)
2016 × Placebo	−0.0345***	(0.0024)
2019 × Placebo	−0.0561***	(0.0030)
2022 × Placebo	−0.0535***	(0.0026)
Controls	Yes	
Region FE	Yes	
Year FE	Yes	
Clusters (NUTS-2)	25	
Observations	124,905	

Source: Türkiye Health Survey Microdata (2008–2022) made available by the Turkish Statistical Institute (TUIK) under a special access agreement. For further details, see https://www.tuik.gov.tr/ (Standard errors in the parentheses)

Notes: Standard errors clustered at the NUTS-2 level. Erzurum (TRA1) is assigned placebo treatment status; the actual treatment region (Van) is excluded. Negative post-period coefficients reflect regional heterogeneity rather than a placebo treatment effect
